# Michigan State Board of Health

**Published:** 1884-06

**Authors:** 


### MICHIGAN STATE BOARD OF HEALTH.

[Reported for the CHICAGO MEDICAL JOURNAL AND EXAMINER.]

The annual meeting of the State Board of Health was held at its office in Lansing, April 8, 1884.

The members present were, President Avery, Drs. Lyster, Hazlewood, Vaughan, Tyler, and Secretary Baker.

Reports of the sanitary condition of the Wayne County Poorhouse and Asylum, and of the Barry County Jail, were made, by the committee who examined these institutions, and these reports were ordered printed.

Dr. Vaughan read a paper prepared by Dr. C. P. Pengra, which gave results of investigations relative to the purification of water by freezing. As a result of a series of elaborate experiments, conducted in the University laboratory, Dr. Pengra found that, contrary to the general impression, freezing does not render water pure. Ordinarily, he found less infusoria and bacteria in ice than in the water from which it was frozen, but the ice contained them in numbers sufficient to preclude its use. In harvesting ice, the greatest care should be taken to get it from a pure source. This valuable paper will be published in the Report of the Board.

The Secretary presented a summary compiled from lists of medical practitioners registered under the new law. The number of counties in the State is 80, from 76 of which reports have been received. The number of registrations returned is 3,285, but in some cases physicians are registered in two or more counties. The number reported to have graduated is 2,351; those who had attended some college but had not graduated, 208; those who had attended no college, 726. The number belonging to all schools reported as having graduated is 72 per cent. of the whole number, the non-graduated collegiates are 6 per cent., and the non-collegiates are 22 per cent. of the whole number. The "graduates" are from all classes of medical colleges, hospitals, medical societies, etc. The number of different "schools" of medicine reported in these sworn statements is about 75, including "Cureopathic," "Indian," etc. In at least one instance it is reported that the sworn statement had to be signed by a "mark," the practitioner being unable to write even his own name.

The number registered as belonging to the four most prominent schools of medicine is as follows: Regular, 1,533; homœopathic, 490; allopathic, 398; eclectic, 366. The proportion of graduates to practitioners is: regular, 87 per cent.; homœopathic, 74 per cent.; allopathic, 81 per cent.; eclectic, 47 per cent. (Of all schools, as above stated, the per cent. is 72).

It was decided to print the names and addresses of the health officers in Michigan as soon as full returns are received. The number in the State is nearly 1,400. A new edition of the document on the prevention and restriction of scarlet fever was ordered, the last edition of 30,000 copies being nearly exhausted. It was also decided to publish facts relative to several outbreaks of trichiniasis in Michigan.

The following resolutions were unanimously adopted:

*Resolved*, That this Michigan State Board of Health respectfully and earnestly memorializes Congress to pass the bill introduced into the House of Representatives, Jan. 8, 1884, by the Hon. Casey Young, or some similar bill, providing for the prevention of the introduction of infectious diseases into the United States, and for procuring information relating to climatic and other conditions affecting the public health.

*Resolved*, That we consider the National Board of Health the best existing and the proper agency to carry on the work mentioned in the preceding resolution.

The Board discussed the merits of several text-books on physiology and hygiene with special reference to the effects of alcohol on the human system, and approved for use in the schools: Martin's "Human Body," briefer course, second edition, containing special chapter on alcohol and other narcotics; and Dr. Eli F. Brown's "Alcohol: Its Effects on Body and Mind."

The amount of office work during the quarter has been large, and has included: the perfecting of arrangements for holding a Sanitary Convention at Hillsdale; preparation of the proceedings of the Ionia Sanitary Convention for the printer; the making of a compilation of the public health laws of Michigan; proof-reading on 96 pages of the Annual Report; issuing blanks for the return of the new health officers in each city, village and township in Michigan; issuing the regular weekly bulletins of meteorology and of sickness in Michigan; the correspondence of the office (postals are not usually copied), covering 750 pages of letter-copying book, of which over 150 pages have direct reference to the prevention and restriction of communicable diseases in Michigan; and the regular computations of data relating to meteorology and sickness.

